# Morphological and semiconductive properties of the anodic oxide layers made on Fe_3_Al alloy by anodizing in tartaric-sulfuric acid mixture

**DOI:** 10.1038/s41598-023-42311-x

**Published:** 2023-09-13

**Authors:** Rubén del Olmo, Magdalena Łazińska, Mateusz Czerwiński, Tomasz Durejko, Marta Michalska-Domańska

**Affiliations:** 1 Institute of Optoelectronics, Military University of Technology, 2 Kaliskiego Str., 00-908 Warsaw, Poland; 2 Institute of Materials Science and Engineering, Faculty of Advanced Technologies and Chemistry, Military University of Technology, Kaliskiego 2, 00-908 Warsaw, Poland

**Keywords:** Engineering, Materials science

## Abstract

It has recently been found that the anodizing of FeAl alloys allows the formation of iron-aluminum oxide layers with interesting semiconducting properties. However, the lack of systematic research on different anodizing regimes is hampering their full exploitation in numerous photoelectrochemical-related applications. This study address, for the first time, the systematic effect of the electrolyte composition on the formation of self-ordered oxide films by anodizing on cast Fe_3_Al alloy. The Fe_3_Al alloy was anodized in 3 electrolytes with different water-ethylene glycol (EG) ratios (pure water, 25 vol.%-EG, and 50 vol.%-EG solutions) at a constant tartaric-sulfuric acids concentration, different voltages (10–20 V) and treatment times (2–60 min). After anodizing, all anodic oxide layers were annealed at 900 °C to form semiconductive iron-aluminum crystalline phases. Conventional techniques were used to systematically ascertain the morphological (SEM/EDS, XRD, eddy-current measurements) and semiconductive (UV–VIS reflectance spectroscopy) properties of these oxide layers. The results confirmed the formation of homogeneous and self-ordered anodic oxide layers at 10 and 15 V, regardless of the electrolyte composition. Namely, anodic films formed in electrolytes containing EG showed lower pore sizes, growth rates, and film thicknesses than those anodic films formed in the aqueous-based electrolyte. The annealing post-treatment results in different Fe-Al oxides (Fe_x_O_y_, FeAl_2_O_4_, etc.) with superior band gap values than those for non-annealed films.

## Introduction

Over the last decade, numerous attempts to maximize hydrogen production have been pursued to reduce the current dependence on fossil fuels and reduce carbon-based emissions^1,2^. So far, the implementation of semiconductive materials with narrow band gap values is one of the most commonly used approaches to reach this ultimate and striving goal. Namely, Fe_2_O_3_ has been identified as a promising material in energy- and photoelectrochemical-related applications due to its low band gap (*E*_g_ ≈ 2.2 eV), high chemical stability, positive valence band edge potential, non-toxicity, and low cost^3–5^.

However, current synthesis methods, such as electrodeposition^6,7^, sol–gel method^6–9^, and chemical vapor deposition^10,11^ involve multiple-step synthesis routes and high economic costs^12^. Among them, anodizing has been targeted as an efficient and cost-effective method to form Fe_x_O_y_-based films on Fe and stainless steel (SS)^13,14^. Notwithstanding, the current drawbacks of these Fe_x_O_y_-based layers include heterogeneities, lack of reproducibility, and a relatively low sunlight absorption efficiency^5,15–18^, thus significantly limiting their application scope.

To avoid these limitations, recent studies are being carried out to form stable and uniform nanostructured anodic oxide films on FeAl alloys^19–22^. This approach may be due to the fact that Al anodizing usually results in self-ordered, homogeneous, and reproducible Al_2_O_3_-based oxide layers^23^. Besides, considering that Fe anodizing is conventionally performed in ethylene glycol (EG)-containing electrolytes^13,14,24^, whereas Al in tartaric-, boric-, oxalic-, phosphoric-sulfuric-based electrolytes^19,23^. Therefore, it may be expected that Al as alloying element may extend the range of electrolytes to promote the formation of more homogeneous and stable anodic films than pure Fe or SS.

So far, the formation of anodic oxides on FeAl alloys has only been reported in sulfuric^22^ and oxalic^20^ acid-based electrolytes. Besides the different morphology compared to Al, a noticeable feature of FeAl anodizing is the higher current densities recorded during the oxide formation, even at low voltages (~ 5–10 V). For instance, anodizing in sulfuric acid at 20 V and 0 °C results in current densities comprised between 3 and 1500 mA cm^−2^ for pure Al and Fe42Al alloy, respectively. This phenomenon may be the reason, to some extent, for the reported low uniformity, poor stability, and partial delamination of the resultant anodic films on FeAl alloys^20,22^. Moreover, during anodizing of FeAl in sulfuric acid solution, depending on the applied voltage the anodic oxide with tunable bandgap form 2.09 eV up to 3.51 eV are formed^22^. These prospective results prompted us to looking for other electrolytes, which could provide us possibilities to obtain anodic oxides with potentially interesting semiconductive properties.

Therefore, this work aims to address for the first time anodizing of Fe_3_Al alloy in a conventional tartaric-sulfuric acid mixture (TSA) with different water-ethylene glycol (EG) ratios (pure water, 25 vol.%-EG, and 50 vol.%-EG solutions) at different voltages (10–20 V) and treatment times (2–60 min). With this electrolyte design and anodizing regime, we expected to achieve significantly lower current density during the anodizing of Fe_3_Al alloy and, consequently, uniform, and self-ordered anodic oxide layers.

Moreover, all as-anodized films were annealed at 900 °C and further characterized to determine the annealing effect in the formation of different crystalline phases with distinct semiconductive properties. The goal of this preliminary study is to ascertain the optimal anodizing regimens to achieve homogeneous anodic films on Fe_3_Al alloy for further photocatalytic and photoelectrochemical studies.

## Experimental part

### Specimens preparation

Fe_3_Al alloy (wt.%: 28.0 Al, 5.0 Cr, 0.08 Zr, 0.04 B, and Fe balance) was obtained by melting the pure elements in a Balzer's vacuum induction furnace and casting them into metal ingots under an Ar atmosphere. Then, the homogenization was performed at 900 °C for 10 h under air atmosphere and, subsequently cooled to room temperature.

Prior to anodizing treatment, Fe_3_Al substrates were cut into a 1 mm-thick layer by the electro-discharge technique. Then, the Fe_3_Al samples were mechanically grounded with SiC paper at different grades (P600, P1200, and P2400) on a STRUERS PLANOPOL 3 machine (Struers Aps., Ballerup, Denmark). Grounded Fe_3_Al samples were electropolished in a Struers ElectroPol device (Struers, Cleveland, OH, USA) with a commercial electrolyte composed of perchloric acid, methanol, and butoxyethanol solution (A3 commercial electrolyte, Struers) at 32 V for 23 s. After electropolishing, all the samples were cleaned in a 1:1 mixture of isopropanol and acetone in ultrasound (CHC-Tech) for 30 min and dried with warm air.

### Anodizing and annealing post-treatment

All studied anodic films were formed in a tartaric-sulfuric-based electrolyte (0.5 M of sulfuric acid and 1 M of tartaric acid, respectively; Sigma-Aldrich) with different amounts of ethylene glycol (0, 25, and 50 vol. %) (Poch™). The anodic film designation, the corresponding operating regimes, and the used electrolytes are summarized in Table 1. Note that a constant temperature (10 °C) and a stirring rate of 300 rpm were used in all treatments. A constant sample area of 1 cm^2^ was used in each experiment. The treatment times were adjusted to have the same applied density of charge. The applied charge in the anodic films formed in 0 vol.%-EG solution at 10 V was used as reference.

**Table 1:** Anodizing regimes and electrolytes used for studied Fe_3_Al alloy anodizing.

Anodic film designation (V)	Electrolyte		Conditions	
	Ethylene glycol [v. %]	H_2_O [v. %]	Voltage [V]	Time [s]
0–10	–	100	10	600
0–15	–	100	15	225
0–20	–	100	20	120
25–10	25	75	10	900
25–15	25	75	15	425
25–20	25	75	20	180
50–10	50	50	10	3600
50–15	50	50	15	1800
50–20	50	50	20	600

The experimental anodizing system was equipped with a 0.5 L double-walled glass cell and connected to a chiller (Huber mini chiller Plus). The chiller was supplied with a commercial coolant (HUBER SynOil, M10.120.08) to maintain the electrolyte temperature at 283 K during anodizing.

All the anodic films were formed on Fe_3_Al alloy (anode) using a DC power supply (SM400-AR-8 Systems electronic) and a pure Pt grid (60 mm diameter, 40 mm long, and 1 mm thick) as a counter electrode (cathode). Note that all the plotted current density-time data from the oxide layers synthesis stem from a representative specimen of five replicas. After anodizing, the samples were rinsed in deionized water and isopropanol and then, dried with warm air.

Ionic conductivity and pH values of all the used electrolytes (Table 2) were measured using a Metler Toledo Inn Lab® instrument equipped with pH (Expert P1O-1SM) and conductivity (731-ISM; C: 10 cm^−1^) probes. All results presented in the manuscript correspond to the mean value and standard deviation of 10 measurements.

**Table 2:** Conductivity and pH values of the studied electrolytes.

Property/electrolyte	0 vol.%-EG	25 vol.%-EG	50 vol.%-EG
σ [mS cm^−1^]	218 ± 5	91 ± 2	44 ± 2
pH	0.58 ± 0.01	0.57 ± 0.01	0.58 ± 0.01

The annealing post-treatment was performed on anodized samples in an air atmosphere (Linn High Term VMK-39-5). The samples were heated at 900 °C with a heating rate of 10 °C min^−1^. Then, once the temperature is achieved, all the anodized specimens were annealed for 2 h and cooled down inside the furnace.

### Anodic films characterization

The morphology and composition of anodized Fe_3_Al specimens were examined in longitudinal view using a field-emission scanning electron microscope FE-SEM (FEI, Quanta 3D, Hillsboro, OR, USA) equipped with EDAX energy dispersion microanalysis (EDS) hardware.

Anodic film thicknesses were determined by an eddy-current meter ISOSCOPE FMP10 (Fischer) equipped with an FTA3.3H probe. ImageJ software was used for image analysis using at least three plan view SEM micrographs per selected anodic film^25^.

Phase identification was carried out by X-ray diffraction (XRD) analysis using a Philips Rigaku Ultima IV instrument (Co Kα = 1.54056 A) operated at 40 kV and 40 mA. XRD patterns were analyzed with ICDD database™. The XRD spectra were acquired from 30° to 80° at a scan rate of 1° min^−1^.

### Band gap acquisition and calculation

To determine the band gap values of annealed and non-annealed anodic films, spectrophotometric analysis was performed using a Lambda 850 UV–Vis instrument (Perkin Elmer) equipped with an integrating sphere.

The Diffuse reflectance spectroscopy (DRS spectra) were recorded over the wavelength range from 200 to 800 nm at room temperature. The optical band gap energies were calculated using spectrophotometry data according to Makuła et al.^26^ (γ = 1/2 (direct band gap)).

## Results and discussion

### Anodic film formation

**Figure 1:**
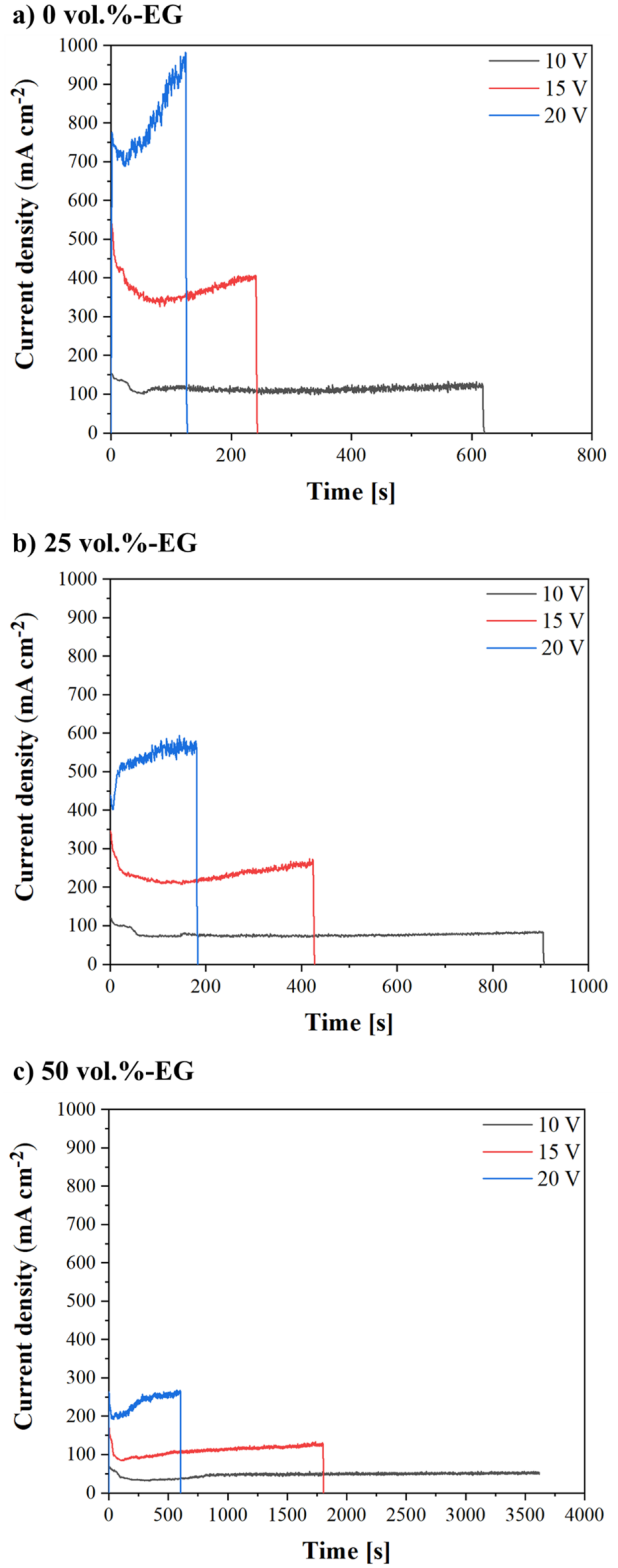
Current density-time curves recorded during Fe_3_Al anodizing in TSA electrolyte with different EG-water ratios (**a**) 0 vol.%-EG, (**b**) 25 vol.%-EG, and (**c**) 50 vol.%-EG.

Figure 1 shows the current density-time responses of the studied Fe_3_Al alloy anodized in TSA electrolyte with different EG-water ratios.

As shown in Fig. 1, all developed anodic films show a characteristic maximum (~ 5 s) in their current–density response. This feature is usually related to the anodic layer growth, oxygen evolution, and dissolution of intermetallic compounds during anodizing^19,27^. Although those are common phenomena in anodizing, note that all anodic films developed in 0 vol.%-EG electrolyte (Fig. 1a) exhibited a constant current increase throughout the treatment, regardless of the applied voltage. This progressive increase in the current–density transient may be associated with the barrier layer thickness and resistivity, i.e., the current density registered for sample 0–20 V reached up to 1000 mA cm^−2^ while for 0–15 V it was around 400 mA cm^−2^ and only 130 mA cm^−2^ for 0–10 V.

By contrast, oxide layers formed in 25 vol.%-EG (Fig. 1b) and 50 vol.%-EG electrolytes (Fig. 1c) showed minimal current density oscillations during anodizing, regardless of the applied voltage. In detail, the maximum current density values for 0–20 V (Fig. 1a) and 50–20 V (Fig. 1c) samples were 1000 mA cm^−2^ and 250 mA cm^−2^, respectively.

This phenomenon is due to the higher EG-water ratio in 25 vol.% EG and 50 vol.% EG electrolytes (Table 2) since it is well-known that the presence of EG in aqueous electrolytes decreases the ionic mobility in the electrolyte, *i.e.*, the electrical conductivity^28^. Additionally, the current density registered for samples made at 20 V highly increase after few first second of reaction. What interesting, the course of current density responds registered for all samples made at 20 V, regardless of the level of EG addition to anodizing electrolyte suggested that for samples made at this voltage the samples burning phenomenon occurs^29^. The burning anodizing is relating to extremely high current flow concentrated at the local points of samples surface, what leading to the local oxide film thickening, for example to formation of cracks or uplifts. In other words, it is the burning of electrolyte at the oxide surface layer without creation of micro-arcs of the growing film^30^. When anodizing is conducted close to or under burning conditions the formation of nanotube dominate on other nano-morphologies, the oxide film contain significantly more species incorporated from the electrolyte solution and the new properties (i.e. change of bandgap or hardness) of such fabricate materials could appear^30^.

Based on the results presented in Table 2, the electrolyte conductivity decreases when the EG content increase as pH values were comparable (same TSA concentration in the studied electrolytes). Accordingly, although the EG concentration in the electrolyte may affect the anodic film growth and charge transfer processes during anodizing, the current density decrease registered during anodizing is more likely related to the formation of a more resistive barrier layer due to the presence of ethylene glycol (EG) in the electrolyte^32–34^.

On that basis, different studies show that the anodizing process on aluminium and iron alloys in the presence of organic additives (e.g., glycolic and oxalic acid) results in the adsorption of these species onto the anodic oxide surface^32,35^. This adsorption process effectively (i) inhibits the chemical dissolution of the oxide layer during anodizing and, (ii) increases the barrier layer resistivity, thus contributing to the observed decrease in current density.

### Characterization of the anodic films

**Figure 2:**
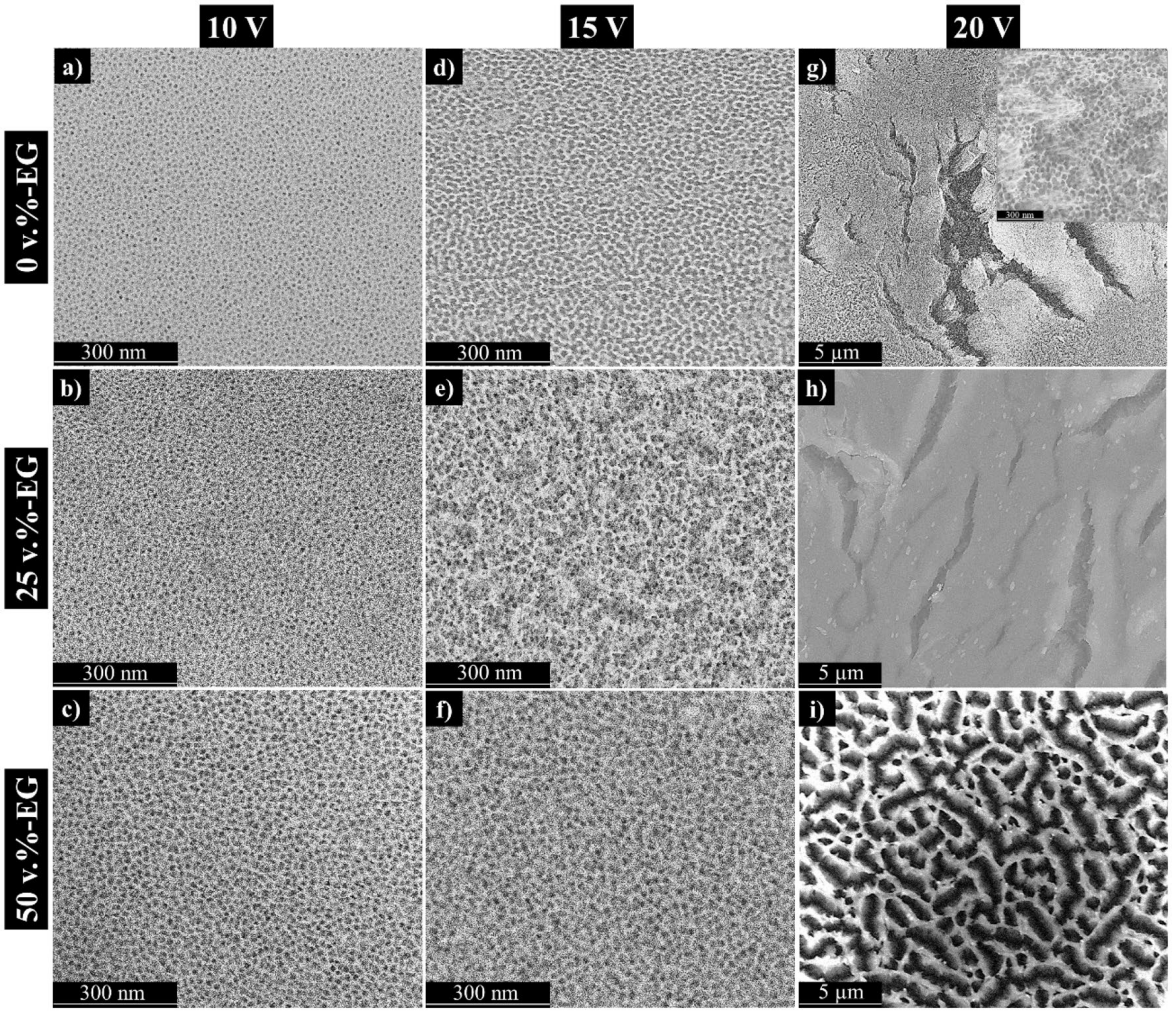
SEM longitudinal views of studied samples at different voltages and EG concentrations.

Figure 2 shows longitudinal SEM micrographs of the studied anodic films formed on the Fe_3_Al alloy. Overall, all anodic films show different surface morphology and thicknesses as a function of the EG-water ratio in the electrolyte and the applied voltage (Table 3).

**Table 3:** Surface features of studied anodic films.

Sample		Porosity [%]	Pore diameter [nm]	Thickness [µm]	Growth rate [µm min^−1^]
0 vol.%-EG	10 V	42 ± 7	12 ± 2	5.2 ± 1.2	0.5
	15 V	62 ± 6	16 ± 3	7.4 ± 1.7	2.0
	20 V	–	22 ± 2	4.2 ± 2.1	2.1
25 vol.%-EG	10 V	56 ± 4	9 ± 1	4.2 ± 0.8	0.3
	15 V	54 ± 8	10 ± 2	6.7 ± 1.8	0.9
	20 V	–	–	3.2 ± 2.2	1.1
50 vol.%-EG	10 V	72 ± 4	10 ± 2	3.8 ± 0.5	0.1
	15 V	64 ± 8	12 ± 2	5.6 ± 1.3	0.2
	20 V	–	–	3.2 ± 2.4	0.3

Regarding the effect of the applied voltage, the anodic layers formed at 10 V (Fig. 2a–c) and 15 V (Fig. 2d–f) show a self-ordered morphology with a variable pore size as a function of the voltage used. As can be seen, the pore diameter is proportional to the applied voltage (Table 3). Although it is well known that the interpore distance and the pore diameter are proportional to the registered voltage, i.e., anode potential, the pore diameter is both influenced by the voltage and the chemical dissolution of the pore walls.

Considering the abovementioned effect of EG on the barrier layer resistivity, it should be noted that the pore diameter values reported in the present study for 0 vol.%-EG electrolyte (Table 3) were comparable to those reported for Fe_3_Al alloys with higher Al content (~ 60–80 at.% Al). Namely, available studies informed that anodizing of these alloys in the electrolyte of 20 wt.% H_2_SO_4_ (aq.) results in pore diameters of 10, 15, and 25 nm at 10, 15, and 20 V, respectively^21,22,35^.

By contrast, results obtained in the present study are in line with the pore size values reported in TSA anodizing of pure Al at similar anodizing conditions (i.e., 10–12 nm at 10 V)^36,37^. These slightly superior pore sizes in Fe_3_Al alloy (Table 3) may be due to the higher Fe concentration in the base material, i.e., higher current densities were reported, and hence, larger pore diameters are prone to be formed. Namely, the registered current density values in conventional TSA of the studied Fe_3_Al alloy are about ten times higher (100–120 mA cm^−2^) (Fig. 1a) than those reported on Al alloys (10–20 mA cm^−2^ at 15–20 V and 20–25 °C in TSA electrolytes)^37–39^.

Although the effect of the applied voltage on anodic oxide morphology is more than evident, the influence of the EG-water ratio in the electrolyte should be mentioned. Fe_3_Al anodizing at 10–15 V in EG-containing electrolytes results in less porous surfaces, lower pore diameters, and thinner films than those formed at 0 vol.%-EG electrolyte at the same voltage range (10–15 V) (Table 3). Again, this may be due to the reduced current density registered during the process, which, in turn, affected the barrier layer resistivity^33,34^ and, to some extent, the decreased ionic migration during anodizing in EG-containing electrolytes^35,40^.

Besides, the loss of homogeneity in the pore distribution is affected by the chemical dissolution equilibrium as well as the burning phenomenon occurs during anodizing at high voltage. Namely, the anodic films formed at 20 V in the all electrolytes shows different morphology compared to samples made at 10 and 15 V. Oxide made at 20 V in EG-free electrolyte showed an underlying porous layer located underneath a homogeneous and compact oxide layer (Fig. 2g). Whan the EG content in electrolyte increased, the registered current density during anodizing at 20 V decreased and the observed cracks on oxide surface become smaller, but there were more of them (Fig. 2h,i). Generally, the morphology of oxide made on Fe_3_Al at 20 V regardless of EG content in electrolyte can be compared with a good approximation to the morphology of anodic aluminum oxide received when the anodizing burning phenomenon take place^41–43^.

Since the pore diameter is not solely dependent on the current density values, it is conceivable that higher current densities in the presence of EG-containing electrolytes may induce a localized temperature increase over the sample surroundings, i.e., the Joule heating effect^44,45^. Although during the anodizing processes, no temperature variations were registered in the electrolyte, this phenomenon may be noticeable considering the small sample size (~ 1 cm^2^) and the high registered current density values.

Therefore, the effect of Joule heating may exert, to some extent, an indirect influence on the chemical dissolution phenomena and, subsequently, on the pore diameter observed in the resulting anodic films (Table 3).

Moreover, the high recorded current densities and the possible joule heating effect may contribute to the increased dissolution rate, leading to the formation of amorphous oxide layer on the top of samples made at 20 V. This may justify, to some extent, the lower coating thickness reported for anodic films formed at 20 V (Table 3; Fig. 2g–i) compared to those developed at 10 and 15 V in all electrolytes.

Interestingly, the anodic films developed in EG-containing electrolytes showed distinct morphologies as a function of the EG-water ratio in the electrolyte. Namely, while the anodic film formed at 25 vol.%-EG electrolyte showed a homogeneous compact layer (Fig. 2h), the anodic film formed at 50 vol.%-EG electrolyte showed a partially sealed morphology (Fig. 2i). This may be justified by the limited mobility of acidic ions in the electrolyte since the presence of EG increases electrolyte viscosity and may hamper the pore formation on the top oxide layer^20^.

Regarding the chemical composition, EDS analysis of the studied anodic films (Table 4) reveals that all the oxides are composed mainly of elements derived from the TSA-based electrolyte (S, C, O) and the substrate (Fe, Al). Since the applied charge is comparable for all formed anodic films and the concentration of Fe and Al in the substrate is also in line, the anodic oxide composition regards the electrolyte composition. The higher C incorporation into the anodic films developed in EG-containing electrolytes may be related to the incorporation of tartrate (C_4_H_4_O_6_^−^) and carboxylate (COO^−^) anions ions from the tartaric and EG solvent into the forming oxide, respectively^46,47^. This organic species incorporation also could affect the phases composition of samples after annealing. Similarly, the S incorporation into the studied anodic films is due to the ${\mathrm{SO}}_{4}^{2-}$ anions migration from the electrolyte to the substrate under the positive electric field during anodizing^27^. Moreover, adsorption of tartrate (C_4_H_4_O_6_^−^) and carboxylate (COO^−^) anions onto the anodic oxide surface may be in line with their effect on the superior barrier layer resistivity and, the consequent current density decreases at higher EG concentrations in the electrolyte^32,46^.

**Table 4:** EDS analysis of the studied anodic films (at%).

Sample		Al	Fe	O	C	S
0 vol.%-EG	10 V	10.6 ± 1.0	15.5 ± 1.2	58.7 ± 4.1	11.8 ± 4.1	3.4 ± 0.2
	15 V	11.2 ± 0.7	16.6 ± 2.3	58.9 ± 2.2	11.4 ± 1.8	1.9 ± 0.2
	20 V	17.2 ± 1.2	11.8 ± 2.2	57.6 ± 3.2	11.1 ± 2.6	2.3 ± 0.1
25 vol.%-EG	10 V	13.3 ± 0.9	17.6 ± 1.1	54.6 ± 1.2	12.3 ± 7.5	2.2 ± 0.4
	15 V	14.2 ± 1.4	16.3 ± 1.6	53.4 ± 4.4	14.0 ± 3.6	2.1 ± 0.2
	20 V	14.3 ± 1.9	12.9 ± 1.7	54.7 ± 7.1	16.2 ± 4.1	1.9 ± 0.6
50 vol.%-EG	10 V	16.3 ± 2.1	19.4 ± 3.3	50.6 ± 3.2	11.3 ± 3.2	2.4 ± 0.3
	15 V	12.5 ± 1.6	16.0 ± 2.7	55.5 ± 4.1	13.0 ± 1.3	3.0 ± 0.3
	20 V	12.3 ± 1.5	11.4 ± 2.5	53.0 ± 4.4	20.9 ± 2.0	2.4 ± 0.3

### Characterization of annealed anodic films

**Figure 3:**
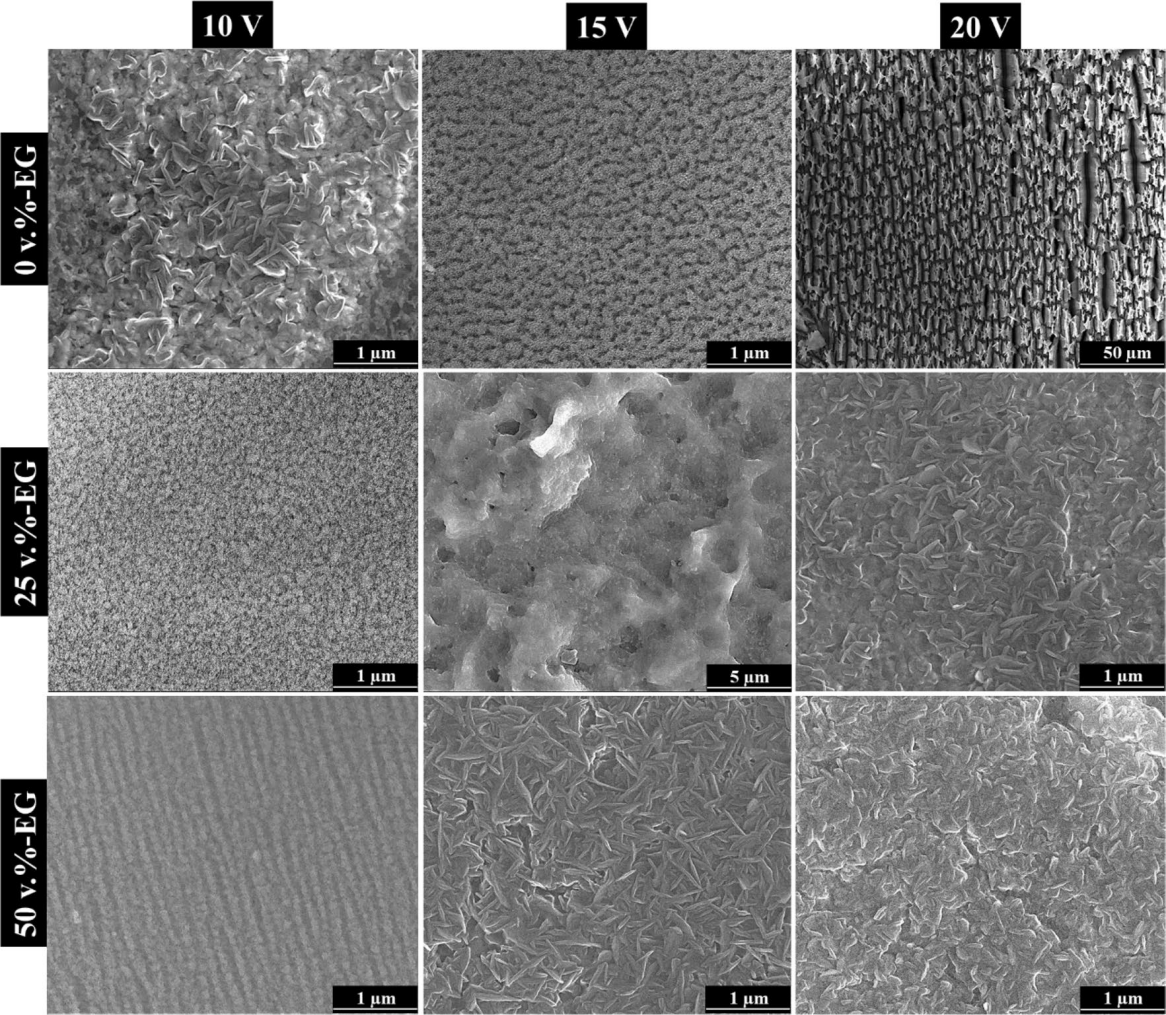
SEM longitudinal views of annealed samples formed by anodizing at different voltages and EG concentrations.

**Figure 4:**
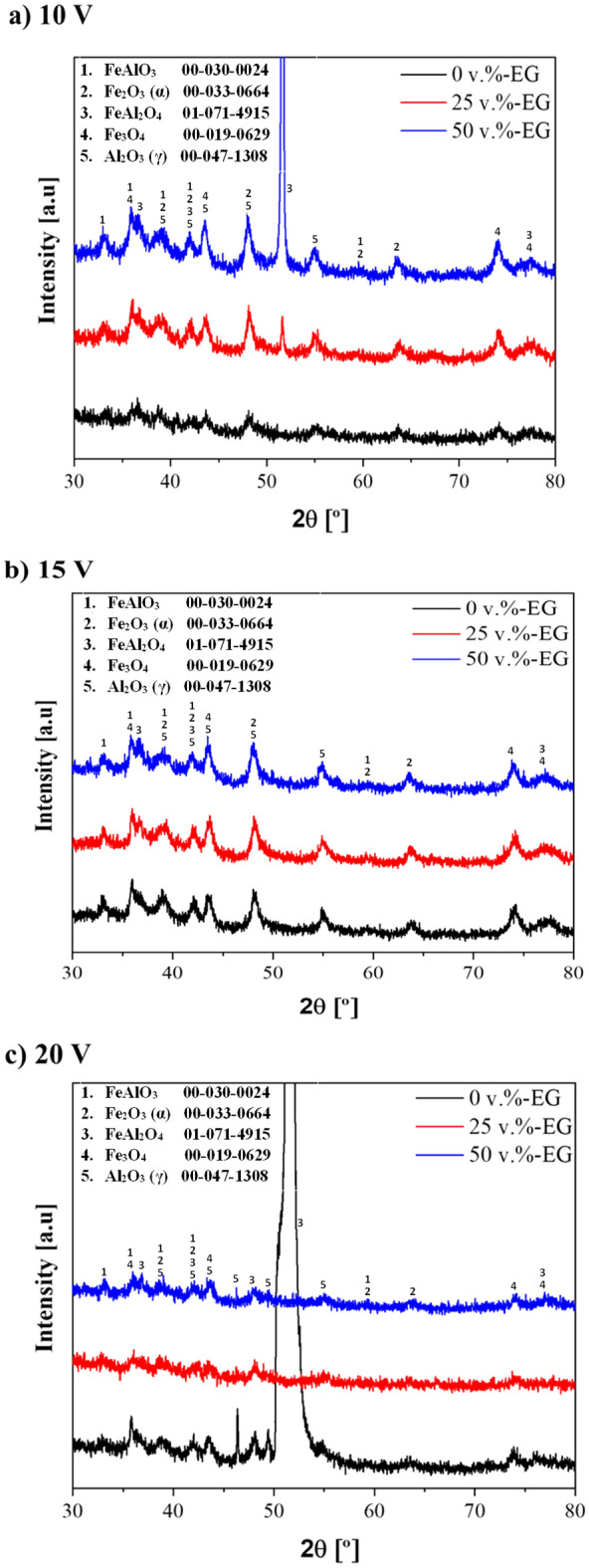
XRD patterns of the studied films developed in (**a**) 0 vol.%-EG, (**b**) 25 vol.%-EG, and (**c**) 50 vol.%-EG electrolytes.

According to the available literature, annealing post-treatment of anodized oxide layers synthesized on FeAl alloys is known to induce the formation of crystalline phase transformations^20,22^. In this study, all the anodic films were annealed at 900 °C for 2 h in air atmosphere to address all possible changes in the surface morphology, chemical composition, and phase transformations. The annealed samples were characterized using scanning electron microscopy (Fig. 3) with energy-dispersive spectroscopy (Table 5) and X-ray diffraction (Fig. 4).

**Table 5:** EDS analysis of the studied annealed anodic films (at.%).

Sample		Al	Fe	O	C
0 vol.%-EG	10 V	30.9 ± 0.8	39.2 ± 1.9	22.6 ± 1.9	7.3 ± 0.9
	15 V	18.2 ± 0.3	20.0 ± 0.6	53.7 ± 0.5	8.1 ± 0.6
	20 V	17.6 ± 0.8	23.4 ± 1.2	51.7 ± 0.9	7.3 ± 0.3
25 vol.%-EG	10 V	19.8 ± 3.3	25.0 ± 7.8	47.8 ± 11.1	7.4 ± 0.1
	15 V	28.3 ± 4.9	32.9 ± 1.2	31.3 ± 4.4	7.5 ± 1.4
	20 V	19.7 ± 0.4	20.3 ± 8.8	51.9 ± 10.0	8.2 ± 0.8
50 vol.%-EG	10 V	17.9 ± 0.4	21.2 ± 5.0	54.2 ± 3.6	6.7 ± 1.8
	15 V	23.6 ± 8.9	27.0 ± 3.3	43.3 ± 10.2	6.1 ± 2.2
	20 V	24.5 ± 8.6	28.3 ± 5.4	40.9 ± 13.8	6.4 ± 0.3

As can be seen in Fig. 3, the surface morphology of the annealed anodic layers exhibited a transition from the nanoporous structure to a compact oxide layer with large and numerous cavities and, in some cases, resembling flakes. Although no clear correlation between electrolyte composition and morphology can be established, several studies reported the occurrence of diffusion processes during annealing. These diffusion phenomena usually lead to the coalescence and fusion of pores, thus resulting in the formation of larger and flatter structures, such as flakes^48–50^.

Furthermore, given the high annealing temperature, it is also plausible that the increased atom mobility in the oxide layer facilitated the reorganization and formation of more compact crystal structures.

Regarding the chemical composition analysis (Table 5), EDS analysis reveals higher contents of iron and aluminum and lower oxygen concentration compared to those from non-annealed films (Table 4). These findings suggest the occurrence of chemical reactions within the oxide layer, i.e., the oxygen from the oxide and the surrounding air atmosphere reacts with the aluminum and iron species^50^.

The higher concentration of iron and aluminum on the surface is in line with the enhanced diffusion of these elements to the surface of the oxide layers^35,49,51^. As a consequence of the annealing temperature (900 °C), the discernible increments in the iron and aluminum concentrations may be presumably attributed to the facilitated diffusion of these elements towards the film surface.

The XRD patterns of all the studied anodic layers after annealing are shown in Fig. 4. Due to their amorphous nature, as-anodized films (before annealing post-treatment) were not included in this analysis. What important, in the case of our study focused on FeAl anodization, the current density was about ten times higher (100–120 mA cm^−2^) than those reported on Al alloys (10–20 mA cm^−2^ at 15–20 V and 20–25 °C in TSA electrolytes)^52^, what could affect phases composition of oxide after annealing.

The presence of crystalline α-Fe_2_O_3_ and Fe_3_O_4_, FeAl_2_O_4,_ FeAlO_3,_ and γ-Al_2_O_3_ phases are observed in all diffraction patterns. Although the presence of Fe_2_O_3_ has been previously reported in anodized iron after annealing at 450 °C in an oxygen atmosphere^13,14,24,46^, the presence of Fe_3_O_4_ is usually negligible at 450 °C. Notwithstanding, Y. Makimizu et al*.*^53^ reported that the Fe_2_O_3_ reduction to Fe_3_O_4_ at 400 °C was associated with low oxygen activity in an Ar atmosphere. In the present study, the presence of Fe_3_O_4_ may be associated with the high annealing temperature (900 °C). Moreover, Fe_2_O_3_ phase may occur in two form: α- (hematite) and γ- (maghemite). Based on available literature above annealing temperature of 700 °C only α-Fe_2_O_3_ can be formed^54–57^, which has been confirmed in our research.

By way of comparison, it is worth mentioning that the peak intensity of Fe_2_O_3_ and Fe_3_O_4_ phases is superior for those films developed at 10–15 V compared to those formed at 20 V, regardless of the used electrolyte (Fig. 4). This may be due to the heterogeneous double-layered morphology of these layers (Fig. 2) since the incorporation of iron into the anodic films is favoured at higher voltage values during anodizing^5,17^.

The presence of Al is also related to the initial formation of amorphous alumina during the anodizing process^58,59^. Aluminum is present in the annealing samples in the form of γ-Al_2_O_3_, which is consistent with the available literature^60–63^.

In phases composition of annealed samples two spinels were detected: FeAlO_3_ and FeAl_2_O_4_. Regarding the presence of FeAl_2_O_4_, several studies reported its formation after annealing at 500–900 °C, especially at higher temperatures^20–22^. In the case of FeAl alloys, several studies reported that the formation and peak intensity of the FeAl_2_O_4_ phase is proportional to the annealing temperature and the voltage applied during anodizing^22,64^. Other study reveals that the FeAl_2_O_4_ in the air atmosphere and at the elevated temperature decompose to Fe_3_O_4_ and γ-Al_2_O_3_^65^. In the present workthe additional FeAl_2_O_4_ peak in the 0–20 V (Fig. 4a) is connected with the high applied voltage during anodizing since the annealing conditions are the same in all the anodic layers^22^. In the case of 50–10 V sample, since the chemical composition after annealing (Table 5) reveals a similar Fe/Al proportion with the other films, the higher intensity of the FeAl_2_O_4_ peak may be due to a different distribution of this phase over the surface film.

### Semiconductive properties of the anodic films

**Figure 5:**
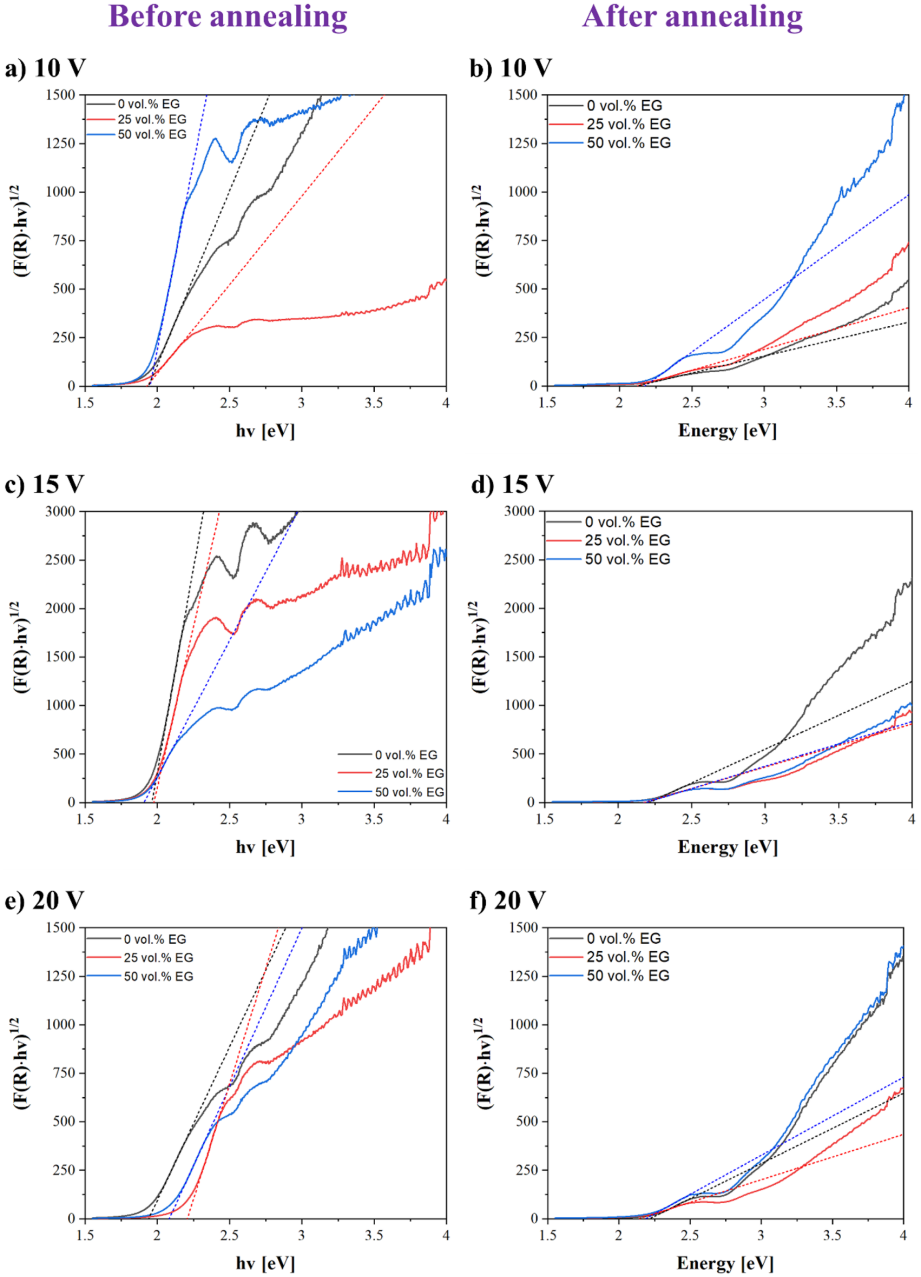
Band gap estimation for selected samples before and after annealing from reflectance measurements.

These different phase proportions are expected to affect the semiconducting properties of the studied as-anodized and annealed oxide films. Figure 5 shows the Tauc plot spectra of selected samples before and after annealing. The band gap values are summarized in Table 6.

**Table 6:** Estimated band gap values of selected samples before and after annealing from UV–Vis measurements.

Anodic film		Band gap^4^		
		Before annealing	After annealing	∆eV
0 vol.%-EG	0–10 V	1.94	2.13	0.19
	0–15 V	1.96	2.22	0.26
	0–20 V	1.94	2.23	0.29
25 vol.%-EG	25–10 V	1.93	2.13	0.20
	25–15 V	1.98	2.19	0.21
	25–20 V	2.20	2.21	0.01
50 vol.%-EG	50–10 V	1.95	2.18	0.23
	50–15 V	1.91	2.20	0.29
	50–20 V	2.08	2.30	0.22

Before annealing, the anodic films formed at 10 V (Fig. 5a; Table 6) and 15 V (Fig. 5c; Table 6) show similar bandgap values (*E*_g_ ≈ 1.94–1.98 eV; ∆*E*_g_ ≈ 0.04), regardless of the EG concentration in the electrolyte. By contrast, the oxide layers formed at 20 V show superior bandgap values in EG-containing solutions, i.e., *E*_g_ ≈ 1.94 eV (0–20 V) *vs. E*_g_ ≈ 2.20 eV (25–20 V) and *E*_g_ ≈ 2.08 eV (50–20 V) (Fig. 5e; Table 6).

This divergence in the band gap values as a function of the applied voltage during anodizing is in line with the study reported by Chilimoniuk et al*.*^64^. The authors studied the morphology, composition, and band gap values of anodized FeAl_3_ alloy in sulfuric acid at different voltages (*E*_g_ ≈ 2.6–2.4 eV for as-anodized films at 10 and 22.5 V). Besides, the authors compared the band gap values with those obtained from anodized FeAl alloy at similar conditions (*E*_g_ ≈ 2.2–2.1 eV for as-anodized films at 5 and 17.5 V)^22^.

The results prove that the oxide layers formed on the alloy with higher aluminium content show superior band gap values than those from the anodized FeAl_3_ alloy^22,64^. However, in the present study, the substrate and the applied charge during anodizing were the same in all tests. Therefore, the observed difference in the band gap values between those samples formed at 10–15 V and 20 V may be associated with the anodizing regime. Namely, although the Al concentration is comparable in all the oxide layers, the higher applied voltage during anodizing may promote the most formation of aluminium oxides^66,67^ with a certain insulating nature (e.g., the band gap of amorphous Al_2_O_3_ is *E*_g_ ≈ 3.2 eV)^68,69^.

After annealing, all the anodic films show higher band gap values than non-annealed films, i.e., ∆*E*_g_ ≈ 0.2–0.3 eV (Fig. 5; Table 6). This is opposite to current findings in the literature since annealing post-treatment of anodized FeAl alloys usually results in crystalline oxide layers with lower band gap values^22,35,64^. This is probably associated with the formation of crystalline and conductive iron oxides, which are usually formed at temperatures above 700 °C.

In the present study, the higher band gap values achieved after annealing (Fig. 5) may be more closely associated with the higher content of Al-containing mixed oxides with a specific insulating nature. For instance, the band gap value of the individual γ-Al_2_O_3_^70^ phase is consistently superior (*E*_g_ is in the range of 8.02–8.7 eV) than those for FeAl_2_O_4_ and Fe_2_O_3_ phases, i.e., *E*_g_ ≈ 3.50 eV and *E*_g_ ≈ 2.1 eV, respectively^53^. This observation is in line with the chemical composition analysis of the annealed anodic layers (Table 5). Specifically, the comparable proportions of aluminum and iron in the annealed samples indicate that the slightly elevated presence of insulating aluminum-containing phases may account for the higher band-gap values of the annealed oxide films.

However, there are slight differences depending on the content of these phases. For instance, in EG-free electrolytes, the band gap difference is more noticeable for samples anodized at 20 V than 15–10 V (Table 6). This may be due to the higher content of FeAl_2_O_4_ and γ-Al_2_O_3_ phases after annealing (Fig. 3a).

By contrast, in EG-containing electrolytes, the oxide layers formed at 20 V show non-proportional band gap values. For 25–20 V and 50–20 V samples, this difference may be associated with their similar morphology (Figs. 2, 3) and the lower Al-containing phases content after annealing (Fig. 4b,c), compared to their analogous formed at 10–15 V^20,22,64^.

## Conclusions

Present findings highlight the relation between anodizing conditions and electrolyte composition for the formation of coherent, self-organized, and uniform layers by anodizing of Fe_3_Al alloy. Namely, the main conclusions from this preliminary study can be summarized as follows:

- The higher concentration of Fe in the base material results in higher recorded current density values than those registered during anodizing of Al by TSA.
- Anodizing of Fe_3_Al alloy in TSA-EG electrolytes at 10 and 15 V results in self-organized oxide films, regardless of the EG concentration. Anodizing of Fe_3_Al at 20 V is anodizing under burning condition and results in the formation of heterogeneous double-layered oxide films.
- The addition of EG to the TSA electrolyte promotes the formation of anodic films on Fe_3_Al with lower porosity, pore diameters, and thickness.
- The annealing post-treatment of the studied anodic films results in anodic films with higher iron and aluminium content and lower oxygen concentration than the as-prepared anodic films.
- Annealing post-treatment results in the formation of α-Fe_2_O_3_, Fe_3_O_4_, FeAl_2_O_4_, FeAlO_3_, and γ-Al_2_O_3_ phases with different proportions.
- Annealing post-treatment at 900 °C results in anodic films with superior band gap values than as-anodized specimens.

## Data Availability

The datasets used and/or analyzed during the current study are available from the corresponding author (M.M.-D.) on reasonable request.
